# Dynamic Responses of Antioxidant and Glyoxalase Systems to Seed Aging Based on Full-Length Transcriptome in Oat (*Avena sativa* L.)

**DOI:** 10.3390/antiox11020395

**Published:** 2022-02-16

**Authors:** Ming Sun, Shoujiang Sun, Chunli Mao, Han Zhang, Chengming Ou, Zhicheng Jia, Yifan Wang, Wen Ma, Manli Li, Shangang Jia, Peisheng Mao

**Affiliations:** Forage Seed Laboratory, College of Grassland Science and Technology, China Agricultural University, Beijing 100193, China; sunmiir@cau.edu.cn (M.S.); b20193040362@cau.edu.cn (S.S.); bs20173040366@cau.edu.cn (C.M.); b20193040364@cau.edu.cn (H.Z.); B20203240983@cau.edu.cn (C.O.); sy20203243205@cau.edu.cn (Z.J.); s20193040624@cau.edu.cn (Y.W.); mmw21@cau.edu.cn (W.M.); lml@cau.edu.cn (M.L.); shangang.jia@cau.edu.cn (S.J.)

**Keywords:** oat, seed aging, antioxidant system, glyoxalase system, dynamic response, full-length transcriptome

## Abstract

Seed aging is a major challenge for food security, agronomic production, and germplasm conservation, and reactive oxygen species (ROS) and methylglyoxal (MG) are highly involved in the aging process. However, the regulatory mechanisms controlling the abundance of ROS and MG are not well characterized. To characterize dynamic response of antioxidant and glyoxalase systems during seed aging, oat (*Avena sativa* L.) aged seeds with a range of germination percentages were used to explore physiological parameters, biochemical parameters and relevant gene expression. A reference transcriptome based on PacBio sequencing generated 67,184 non-redundant full-length transcripts, with 59,050 annotated. Subsequently, eleven seed samples were used to investigate the dynamic response of respiration, ROS and MG accumulation, antioxidant enzymes and glyoxalase activity, and associated genes expression. The 48 indicators with high correlation coefficients were divided into six major response patterns, and were used for placing eleven seed samples into four groups, i.e., non-aged (Group N), higher vigor (Group H), medium vigor (Group M), and lower vigor (Group L). Finally, we proposed a putative model for aging response and self-detoxification mechanisms based on the four groups representing different aging levels. In addition, the outcomes of the study suggested the dysfunction of antioxidant and glyoxalase system, and the accumulation of ROS and MG definitely contribute to oat seed aging.

## 1. Introduction

Seed aging is inevitable during seed storage, and results in the decrease of seed emergence rate, seedling consistency, and field resistance, which thereby are strongly related to agronomic production, food security, and germplasm conservation [1,2]. Genetic and environmental factors are involved in seed aging, and often lead to a series of changes in physicochemical and physiological properties. While the mechanism of seed aging has attracted much attention for quite some time, there are still many hypotheses at present, such as attacks of accumulated ROS, effects of toxic substance, and disfunction of cellular structures and macromolecules [3,4,5,6]. Therefore, a comprehensive understanding of the mechanisms of toxicity and detoxification during seed aging will be beneficial to the improvement of seed vigor.

Generally, accumulation of ROS has been considered to be among the foremost explanations for aging, because it damages cellular components, including nucleic acids, lipids, and proteins [7,8]. In orthodox seeds, the mitochondrial electron transport chain (mtETC) is the main source of ROS, except in the early developmental phase [8]. Superoxide (O_2_·^−^) from mtETC can be converted to hydrogen peroxide (H_2_O_2_), which has a strong oxidizing capacity and long-life span [9]. In the plant antioxidant defense system, superoxide dismutase (SOD) grants frontline protection against ROS by converting O_2_·^−^ to H_2_O_2_. Subsequently, catalase (CAT), peroxidase (POD), and Ascorbate-Glutathione (AsA-GSH) cycle transform H_2_O_2_ to H_2_O [10]. GSH and AsA are present in cellular organelles in a millimolar range, which can directly scavenge ROS or act as electron donors to ROS-processing enzymes [10]. AsA and ascorbate peroxidase (APX) convert H_2_O_2_ into H_2_O and monodehydroascorbate (MDHA), which could be spontaneously transformed into dehydroascorbate (DHA). Meanwhile, with the help of GSH, DHA could be reduced back to AsA, and oxidized glutathione (GSSG) is produced due to oxidation of GSH. In addition, glutathione reductase (GR) and monodehydroascorbate reductase (MDHAR) could regenerate AsA and GSH using NAD(P)H as the electron donors [10]. AOX pathway can also play a significant role in maintaining electron flow in the mtETC and reducing ROS production under adverse conditions [6,7]. Additionally, anaerobic respiration, which often occurs during the seed storage and early germination stages, is also a protective strategy against ROS [11]. In this process, lactate dehydrogenase (LDH) is the key enzyme which is composed of two subunits encoded by the *LDHA* and *LDHB* genes, respectively. In terms of resistance to oxidative damage, the AOX pathway and anaerobic respiration might be active in maintenance of seed vigor. Previous works have shown the considerable role of an antioxidant system in seed aging. Where, for instance, the mutants lacking of glutathione reductase 2 (GR2), dehydroascorbate reductase (DHAR1), and thioredoxin-o1 (Trx-o1) in *Arabidopsis* exhibited reduced seed vigor after aging treatment [12,13]. Simultaneous over-expression of the *CuZnSOD* and *APX* genes in plastids improves tobacco (*Nicotiana tabacum*) seed longevity and germination under various environmental stress conditions [14]. In addition to oxidative damage, as a signal molecule in plants, ROS could crosstalk with other aging response regulators, especially mitogen-activated protein kinase (MAPK) cascades and various transcription factors (TFs). These molecules are closely related to seed vigor [13,15]. Therefore, it is necessary to comprehensively explore the response mechanisms of an antioxidant system on seed aging.

Similar to ROS, methylglyoxal (MG) is also a potential cytotoxin capable of complete disruption of cellular functions at high concentration, and is now emerging with signal molecule function at a low concentration in plants [16,17]. As a reactive dicarbonyl compound, MG can spontaneously glycate nucleic acids, proteins, and lipids, by forming advanced glycation end products (AGEs). Extensive studies have indicated that aldehydes can be accumulated during seed storage, and subsequently inhibit germination [18,19]. Additionally, exogenous MG directly inhibits germination and root elongation [20]. The primary route for detoxification of MG is the glutathione-dependent glyoxalase system, a two-step enzyme-catalyzed pathway, including glyoxalase I (S-D-lactoylglutathione lyase, GLX1) and glyoxalase II (hydroxyacylglutathione hydrolase, GLX2). The spontaneous reaction between GSH and MG forms hemithioacetal which is converted to S-D-lactoylglutahione (SLG) by GLX1. Then, SLG is converted to D-lactate and GSH by GLX2 [21]. The abundant functions of glyoxalases has been reviewed, including regulation of seed germination and viability, cell death, cell signaling, and aging process [17]. However, it is worth noting that the roles of MG and glyoxalases in seed aging have not been systemically reported so far.

Oat (*Avena sativa* L.) is the sixth important cereal crop in the world, and contains high health-promoting components, such as lipids, proteins, dietary fibers, antioxidants, and vitamin B, as well as avenanthramides [22,23,24]. Also, it is probably the most widely used cool-season annual forage [25]. However, compared to the other major crops, the lack of genomic data is one of the crucial problems for conducting genetic improvement in oat. Recently, single-molecule transcriptome sequencing has become a suitable tool for investigating full-length transcripts and constructing reference gene sets using the PacBio RS system [26]. It can sequence both the 5′ and 3′ untranslated regions and the poly (A) tails of cDNA, as well as overcome the limitations of short-read sequences. In addition, this method provides high efficacy for identifying alternative isoforms, alternative polyadenylation, long non-coding RNA (lncRNA), and expressed sequence tag (EST)-simple sequence repeat (SSR) markers [26,27,28]. Based on full-length transcriptomes, pathways related to tissues development and secondary metabolism were better understood [29,30,31]. Therefore, it is feasible and innovative to explore the toxicity and detoxification mechanism of oat seed aging based on the full-length transcripts.

Overall, a robust, well-annotated hexaploid oat full-length transcriptome is very essential for genetic improvement of the major agronomic traits. In addition, the comprehensive involvement of the antioxidant and glyoxalase system in oat seed aging is hardly reported. Hence, the major objectives of this study were (a) to construct a full-length cDNA library from multiple tissues of a hexaploid oat using the PacBio RS system; (b) to investigate the physiological response dynamic patterns of antioxidant and glyoxalase systems; (c) to investigate the dynamic response patterns of genes related to antioxidant and glyoxalase systems; (d) to propose an antioxidant and glyoxalase response model for oat seeds aged for different levels.

## 2. Materials and Methods

### 2.1. Preparation of Seeds

Oat (cv Challenger) seeds obtained from BrettYoung company (Winnipeg, MB, Canada) were harvested in 2018. At the beginning of the test in June 2019, the moisture content of a seed sample was 7.9% (on a fresh-weight basis), and germination percentage was 100%. Seeds with uniform size and plumpness were obtained by using a sieve for further experiments.

### 2.2. Materials for PacBio Sequencing

**Figure 1:**
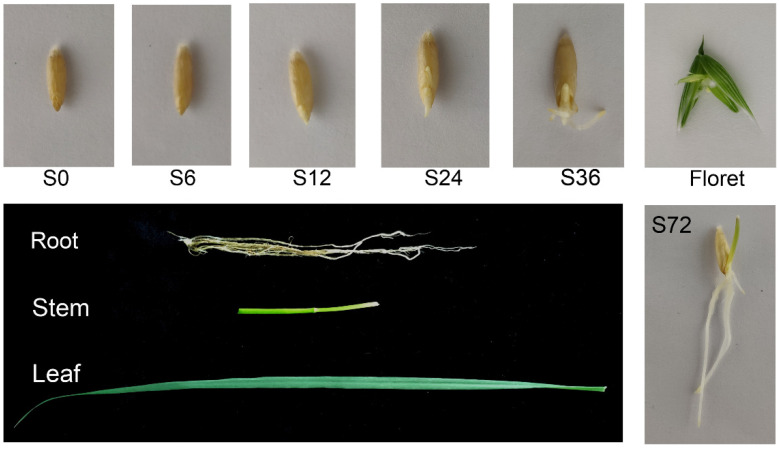
Tissue samples used for oat PacBio sequencing. Embryos samples were collected from oat seeds imbibed for 0 h (S0), 6 h (S6), 12 h (S12), 24 h (S24), and 36 h (S36). Oat seeds imbibed for 6 h (S6) and 24 h (S24) were used to collect endosperms. Seedlings were obtained from oat seeds imbibed for 72 h (S72). Roots, stems, leaves, and florets samples were collected from flowering plants.

The oat seeds were cultured to obtain seven tissue samples, including embryos, endosperms, seedlings at germination stage, leaves, stems, roots, and florets on the day of blooming (Figure 1). Twenty embryos obtained from seeds imbibed for 0 h (S0), 6 h (S6), 12 h (S12), 24 h (S24), and 36 h (S36), respectively, were pooled as embryo sample. The same number of endosperms at 6 h (S6) and 24 h (S24) after imbibition were pooled as an endosperm sample. The seedling sample was a pool of 10 seedlings after imbibition for 72 h (S72). On the day of flowering, stems, roots, leaves, and florets were collected from 10 healthy plants. The samples collected were immediately frozen in liquid nitrogen and stored at −80 °C until further use.

### 2.3. RNA Preparation, Library Construction, Sequencing, and Iso-Seq Data Processing

Total RNA of seven tissue samples was extracted using Quick RNA isolation Kit (Huayueyang Biotech Co., Ltd., Beijing, China). Before library construction, quality and integrity of total RNA were determined using the Agilent 2100 Bioanalyzer (Agilent Technologies, Santa Clara, CA, USA). A Nanodrop 2000 spectrophotometer (Thermo Fisher, Waltham, MA, USA) was used to measure the RNA concentration, and samples with more than 200 ng/uL and optical density (OD) 260/280 above 2.0 were used.

To construct PacBio Iso-Seq library, total RNA from embryo, endosperm, seedling, leaf, stem, root, and floret were mixed with a ratio of 2: 1: 1: 1: 1: 1: 1. The Iso-Seq protocol was performed on a PacBio sequencer using the RSII platform, as previously described [32]. The raw reads in FASTA format were initially filtered with a read accuracy value of less than 0.90 and a read length value of less than 50 bp. The clean reads were processed to generate circular consensus (CCS) reads (run settings: minimum of 1 subread at 90% CCS read accuracy). After examination for the poly (A) signal and 5′ and 3′ adaptors, only the CCS reads with all three signals were considered as a full-length non-chimeric (FLNC) read. The described FLNC reads were clustered by using Iterative Clustering for Error Correction software to generate the cluster consensus with high-quality isoforms (over 99% accuracy). Finally, redundancies were removed to obtain the final set of non-redundant full-length transcripts by CD-hit [33]. The full-length transcripts were compared against public databases, including NCBI non-redundant protein database (Nr), Protein Family (Pfam), Clusters of Orthologous Genes (COG), EuKaryotic Orthologous Groups (KOG), evolutionary genealogy of genes: Non-supervised Orthologous Groups (egg-NOG), Kyoto Encyclopedia of Genes and Genomes (KEGG), and Gene Ontology (GO) using BLAST software (version 2.2.26).

### 2.4. Identification of LncRNA, TFs and EST-SSRs

Four different tools, Coding-Potential Assessment Tool (CPAT) [34], Coding-Non-Coding Index (CNCI) [35], PfamScan and Coding Potential Calculator (CPC) [36] were used to calculate the ability of transcripts to encode proteins. Transcripts longer than 200 bp and more than two exons were selected as lncRNA candidates. TFs in the transcriptome were also identified by conducting a search of the Plant Transcription Factor Database (PlnTFDB, v3.0; http://plntfdb.bio.uni-potsdam.de/v3.0/, accessed on 22 February 2020). Because oat was not included in the database, the TFs were identified through hmmsearch based on the Protein Family (Pfam) search results of the TF family. MISA (MIcroSAtellite identification tool; http://pgrc.ipk-gatersleben.de/misa/, accessed on 22 February 2020) was used to detect potential SSRs from the transcripts. The unit sizes and their minimum number of repetitions were set to default parameters: 110, 2-6, 3-5, 4-5, 5-5, and 6-5. For example, 6-5 indicates that hexa-nucleotide types should have more than 5 repetitions.

### 2.5. Aging Treatment

Aging treatment was performed according to Xia et al. [4]. Briefly, seeds pre-adjusted to 10% moisture content on a fresh-weight basis were immediately sealed in an aluminum foil bag (0.12 × 0.17 m^2^, approx. 40 g in each bag) at 45 °C in a water bath. After 20 d treatment, samples began to be taken out every two days. The germination percentage declined to 93% on the 24th day and decreased to 0% on the 40th day. Seeds treated from the 24th day to the 42nd day with a range of germination percentages were obtained for subsequent tests, and marked as D24, D26, D28, D30, D32, D34, D36, D38, D40, and D42, respectively. The control seeds were marked as D0.

### 2.6. Germination Parameters Tests

Germination tests were conducted according to International Seed Testing Association (ISTA, 2018) criteria, and 3 replicates were used, around 50 seeds per biological replicate. A seed was considered to be germinated if it had developed into a normal seedling. Germination percentage was the normal seedling percentage at the final day (day 10). Leaf length (LL), root length (RL) and seedling fresh weight (FW) were also measured on 10th day. Seeds with primary roots of at least 2 mm long were recorded every day until the 10th day to calculate mean germination time (MGT) using the Equation (1):

MGT = ∑NT/∑N(1)

Vigor index (VI) was calculated according to the Equation (2):

VI = ∑ (G_T_/T) × FW (2)

where T is the number of days counted from the beginning of germination, N is the number of seeds germinated on day T, and G_T_ is the number of germinated seeds per day corresponding to T [37].

### 2.7. Respiratory Measurement

Respiratory consumption of O_2_ were measured using a Clark-type oxygen electrode at 25 ± 1 °C in a 2 mL measuring volume. Seeds imbibed for 24 h were used as the samples. For each measurement, 5 seeds were added in 2 mL of respiration buffer (50 mM HEPES, 10 mM MES, 0.2 mM CaCl_2_, pH 6.6). The oxygen consumption was recorded for 10 min each run using Oxytrace Plus software (Hansatech Instruments Ltd, Norfolk, UK), and the linear part was used to calculate oxygen consumption rate (OCR).

### 2.8. Measurements of H_2_O_2_, MDA, and MG

The embryo samples (0.2 g) from seeds imbibed for 24 h were ground into powder using liquid nitrogen. To measure malondialdehyde (MDA), samples were homogenized with 5% (*w*/*v*) trichloroacetic acid (TCA), and mixed with equal volume of 0.5% thiobarbituric acid. The homogenate was incubated at 95 °C for 30 min and centrifuged at 16,000× *g* for 20 min [38]. The content of MDA was calculated by measuring absorbance at 532 nm and 600 nm. H_2_O_2_ levels were determined according to Velikova et al. [39]. Samples were homogenized in ice bath with 0.1% (*w*/*v*) TCA. The homogenate was centrifuged at 12,000× *g* for 15 min and 0.5 mL of the supernatant was added to 10 mM phosphate buffer (pH 7.0) and 1 mL 1 M KI. The absorbance of supernatant was recorded at 390 nm. Methylglyoxal assay was according to the method of Yadav et al. [40]. About 0.2 g embryo samples was extracted in 2 mL of 0.5 M perchloric acid. The reaction mixture contained 7.2 mM 1,2-diaminobenzene, 5 M perchloric acid, and 650 µL of the neutralized supernatant. The absorbance of the derivatized MG was recorded immediately at 336 nm for 1 min.

### 2.9. Measurements of Antioxidant Enzyme and Glyoxalase Activities

Embryos from seeds imbibed for 24 h were used for enzyme activities estimation. For protein extraction, isolated embryos (0.2 g) were ground in liquid nitrogen and suspended in homogenization buffers which were composed of 50 mM phosphate buffer (pH 7.0) with 1.0 mM EDTA and 1% PVP. After centrifugation at 4 °C for 20 min at 12,000× *g*, supernatants were collected and used to determine enzymes activity. SOD activity was assayed by measuring its ability to inhibit the photochemical reduction of nitroblue tetrazolium, and 50% suppression of the reaction was considered as one enzyme unit [41]. CAT activity was measured by monitoring the decrease of absorbance at 240 nm for 1 min due to the decline of H_2_O_2_ extinction according to the method of Hossain et al. [42]. The activity of POD was measured using guaiacol and hydrogen peroxide as substrates, and the absorbance changes of reaction mixture at 470 nm were determined [43]. GR activity was measured by calculating the decrease in absorbance at 340 nm due to NADPH oxidation [42]. MDHAR activity was measured by detecting the oxidation rate of NADH at 340 nm [42]. The APX activity was determined following the depletion in absorbance at 290 nm due to AsA consumption [44]. DHAR was measured by the increase of absorbance at 265 nm [44]. Total protein concentrations were measured using the Bradford method.

GLX1 and GLX2 assay was carried out using the method of Hossain et al. [45]. The GLX1 reaction mixture contained 100 mM phosphate buffer (pH 7.0), 15 mM MgSO_4_, 1.7 mM GSH, and 3.5 mM MG, and the increase in absorbance was recorded at 240 nm once MG was added. The GLX2 reaction mixture contained 100 mM Tris-HCl buffer (pH 7.2), 0.2 mM DTNB, and 1 mM S-D-lactoylglutathione, and the activity was measured by monitoring the formation of GSH at 412 nm.

### 2.10. Measurements of Non-Enzymatic Antioxidants

Embryos from seeds imbibed for 24 h were used for measurements of non-enzymatic antioxidants. The contents of AsA, DHA, GSH, and GSSG were determined using the commercial assay kits of ASA-2A-W, DHA-2-W, GSH-2-W, and GSSG-2-W, respectively, procured from Suzhou Comin Biotechnology Co., Ltd., China. About 0.1 g samples were extracted in 1 mL extract solution in accordance with the instructions of manufacturer (http://www.cominbio.com/, accessed on 26 December 2021). AsA was extracted with acetic acid solution, and DHA, GSH, and GSSG were extracted with metaphosphoric acid solution. The determination of AsA relies on the reaction of AsA with the Fast Blue B salt, and absorbance was measured at 420 nm. The content of DHA was measured by detecting the rate of reducing DHA to ASA after adding dithiothreitol (DTT) in absorbance at 265 nm [46]. GSH assay is based on sequential oxidation of GSH by DTNB and reduction by NADPH in the presence of GR, and GSH content was determined at 412 nm based on enzyme recycling [45]. GSSG content was determined by using 2VP as the GSH masking agent, according to the previous method [47].

### 2.11. Gene Expression Analysis

The aged seed samples were imbibed for 24 h, and their embryos were collected for total RNA isolation using Quick RNA isolation Kit (Huayueyang Biotech Co., Ltd., Beijing, China). The first-strand cDNA was synthesized, and quantitative RT-PCR was performed in a CFX96 Real-Time System (Bio-Rad, Hercules, CA, USA) with the *AsEIF4A* gene as internal control [48]. Three biological replications were performed. The PCR conditions were as follows: 95 °C for 25 min, and 40 cycles of 95 °C for 5 s and 60 °C for 10 s. Normalized transcript levels were calculated using the comparative CT method. Primers of genes (obtained from PacBio sequencing) used for quantitative RT-PCR are listed in Table S1.

### 2.12. Data Analysis and Figure Construction

Significant differences between aging treatments were calculated in SPSS Statistics 22 using ANOVA and a Duncan’s test. Pearson’s correlation coefficients and correlation heatmap of 48 indicators were obtained by using R packages “corrplot” and “pheatmap”. To show the response patterns of 48 indicators, the data was transformed into Z-scores using SPSS Statistics 22. The means and standard errors were computed, and bar charts and line charts were constructed using GraphPad Prism version 8.0. The UPGMA and Euclidean distance-based cluster analysis were performed using PAST version 3.02.

## 3. Results

### 3.1. Construction of Full-Length Transcriptome

**Figure 2:**
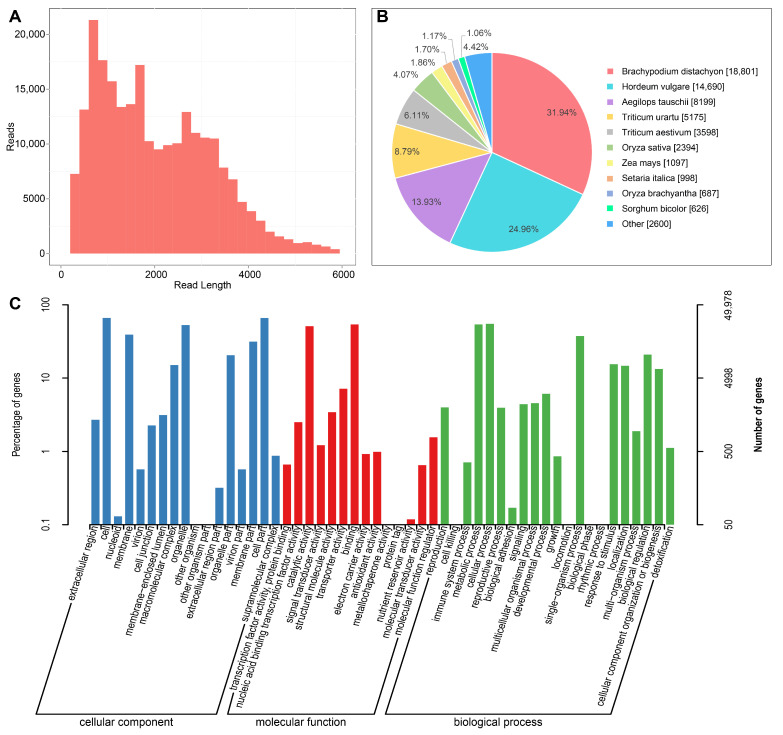
Construction of oat full-length transcriptome through PacBio sequencing. (**A**) CCS read length distributions. (**B**) Homologous species distribution of oat annotated in the Nr database. (**C**) GO functional classification of transcripts.

A total of 293,642 circular consensus sequences (CCSs) were obtained and distributed from 0 to 6000 nucleotides (nt) with an average length of 2209 nt (Figure 2A). And 244,868 (83.39%) full-length non-chimeric (FLNC) reads were generated from the CCSs. The FLNC reads were clustered into 107,320 consensus isoforms with an average length of 2094 nt, of which 103,732 were polished for high-quality isoforms. Finally, 67,184 non-redundant full-length transcripts were generated by using CD-hit software (Table 1).

The full-length transcripts were annotated by aligning against a variety of protein databases (Nr, Pfam, COG, KOG, egg-NOG, KEGG, and GO). The best transcript was selected from the matches with an E-value of less than 10^−5^. In total, 59,050 transcripts were annotated in the seven databases, including 58,923 transcripts in Nr, 57,492 transcripts in egg-NOG, 49,978 transcripts in GO, 42,405 transcripts in Swiss-Prot, 36,286 transcripts in KOG, 25,876 transcripts in KEGG, and 24,110 transcripts in COG. Analysis of Nr homologous species distributions showed that oat is closely related to other Poaceae plants (Figure 2B), such as *Brachypodium distachyon* (31.94%), *Hordeum vulgare* (24.96%), *Aegilops tauschi* (13.93%), and *Triticum urartu* (8.79%).

GO enrichment analysis was carried out to classify the gene functions of the identified full-length transcripts (Figure 2C). A total of 247,492 transcripts were assigned to 52 GO terms of three categories (biological process, molecular function, and cellular component). Biological process containing the most genes (94978) was the largest category, in which “cellular process”, “metabolic process”, and “single-organism process” were identified to be the top three abundant GO terms. Also, “catalytic activity” and “binding” dominated the molecular function category, while “cell”, “cell part”, and “organelle” were the most parts in the biological process.

According to the coding potential of transcripts, we predicted 4352, 11,429, 10,183, and 18,222 sequences as non-coding RNAs by CNCI, CPAT, CPC, and Pfam, respectively. Among them, 3332 sequences were predicted by all four programs, and these transcripts were considered as lncRNAs (Figure S1A). A total of 2164 transcripts were predicted to be TFs, among which 146 transcripts were found in the biggest family of bZIP. The top 20 TF families were shown in Figure S1C, for example, MYB-related, AP2/ERF-ERF, bHLH, C2H2, C3H, MYB, GRAS, and WRKY families. Additionally, 63,032 sequences with a read length of more than 500 bp were examined to identify SSRs using MISA. The result showed 19,281 SSRs were identified from 14,940 sequences, among which 3183 sequences contained more than one SSR. Analysis of SSR types showed that the numbers of mono-nucleotide, di-nucleotide, tri-nucleotide, tetra-nucleotide, penta-nucleotide, hexa-nucleotide, and compound SSRs were 4928, 3894, 9660, 571, 113, 115, and 1387, respectively (Figure S1B).

### 3.2. Effect of Aging Treatment on Seed Germination Characteristics

**Figure 3:**
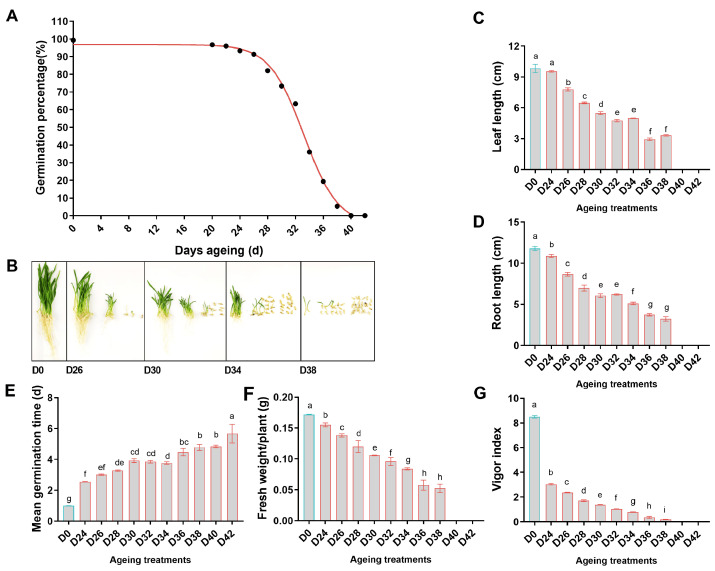
Effect of aging treatments on oat seed germination and seedling growth. (**A**) The declines in the percentages of seeds capable of producing normal seedlings. (**B**) Seeds and seedlings after 10-days germination. (**C**–**E**) Effect of aging treatments on seedling growth. (**F**,**G**) Effect of aging treatments on mean germination time and vigor index. D0 represents untreated control seeds. D24–42 represent seeds with 24 to 42 d ageing treatments. The letters represent statistical significance among the treatments and the vertical bars represent the ±SEM at *p* < 0.05 level for three replicates. The mean values sharing same letters, obtained from Duncan test, are not different significantly.

Aging treatments on oat seeds obtained a range of seed samples with initial germination percentage of 100% (D0) to 0% (D40 and D42). Germination percentages began to decrease after 24 d of aging, and then declined rapidly (Figure 3A,B). The leaf length, root length, and fresh weight of the 10th-day seedlings decreased gradually with the duration of aging (Figure 3C,D,F). MGT was calculated to estimate germination rate. The MGT of aged seeds needed more than 48 h compared with 24 h of non-aged seeds, and the MGT of aged seeds increased with aging duration (Figure 3E). On the contrary, the VI dropped rapidly after seed aging for 24 d (Figure 3G). In short, aging treatments decreased oat seed vigor, which was manifested as slower germination rate, smaller seedlings, and reduced number of normal seedlings.

### 3.3. Effect of Aging Treatment on Respiration, H_2_O_2_ Accumulation, and Lipid Peroxidation

**Figure 4:**
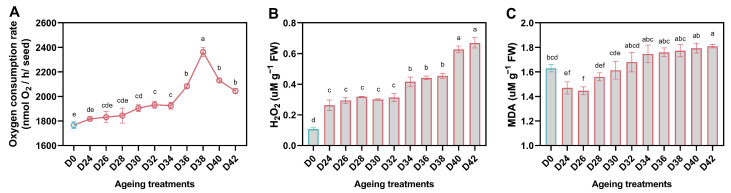
Influence of aging treatment on the oxygen consumption rate (**A**), H_2_O_2_ accumulation (**B**) and MDA content (**C**) of oat seeds. The letters represent statistical significance among the treatments and the vertical bars represent the ±SEM at *p* < 0.05 level for three replicates. The mean values sharing same letters, obtained from Duncan test, are not different significantly.

We measured OCR for the oat seed samples which were imbibed for 24 h, and found the OCR of aged seeds (D24-D42) was significantly higher than that of control seeds (D0). After aging treatment, OCR increased gradually, peaked in D38, and then decreased. The decrease of oxygen consumption capacity in D40 and D42 might contribute to the failure of normal germination (Figure 4A). The result also indicated that aged oat seeds needed more oxygen to sustain germination.

Unsurprisingly, the level of H_2_O_2_ in the aged oat seeds gradually increased after aging, and the content in D24 was more than twice that of D0. D40 and D42 without normal seedlings formation showed the highest H_2_O_2_ level (Figure 4B). MDA is commonly known as a marker of oxidative stress. Our result also showed an upward trend of MDA in aged seeds, which was consistent with the H_2_O_2_ change. But the content of MDA in D0 was higher than D24 and D26, which suffered a relatively minor aging treatment (Figure 4C).

### 3.4. Physiological Changes in Antioxidant System during Aging

**Figure 5:**
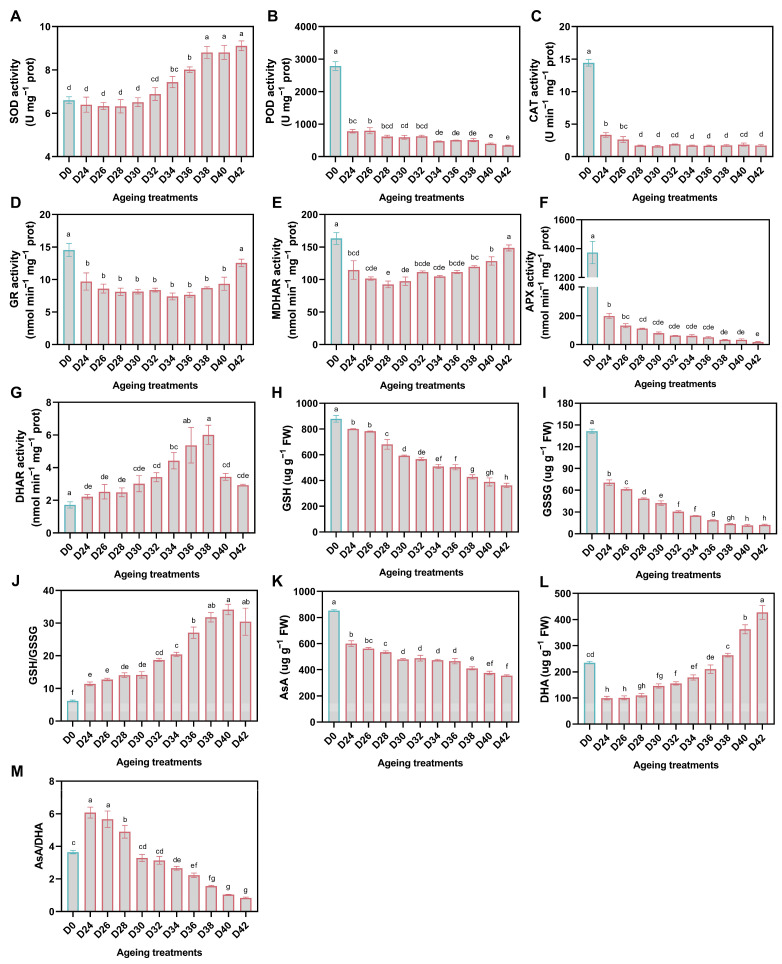
Effect of aging treatment on the activity of antioxidant enzymes (**A**–**G**), the content of non-enzymatic antioxidants (**H**,**I**,**K**,**L**), and the ratio of reduced and oxidized antioxidants (**J**,**M**) in oat seeds. The letters represent statistical significance among the treatments and the vertical bars represent the ±SEM at *p* < 0.05 level for three replicates. The mean values sharing same letters, obtained from Duncan test, are not different significantly.

With aging treatment duration, CAT, POD, and APX activities decreased sharply once germination percentage decreased, while SOD activity increased progressively. (Figure 5A–C,F). GR, MDHAR, and DHAR act on GSH and AsA regeneration in AsA-GSH cycle. The activity of GR and MDHAR firstly decreased and then increased with the aging duration (Figure 5D,E). Whereas, DHAR activity gradually increased, peaked in D38, and decreased in D40 and D42 (Figure 5G). The content of both GSH and its oxidized form GSSG showed a downward trend, and GSSG decreased more considerably after 24 d aging treatment compared to GSH. Interestingly, the ratio of GSH/GSSG increased with aging time, and maintained high levels in D38, D40, and D42 treatments (Figure 5H–J). The changes of AsA content also decreased with aging time, and were similar to GSSG. The amount of DHA increased from D24 to D42, while D0 exhibited a medium level, and its content was significantly lower than that of severe aged treatments. In aged seeds, the ratio of AsA/DHA decreased approximately 6-fold from D24 to D42, and showed a medium level in D0 (Figure 5K–M).

### 3.5. Changes in MG Content and Glyoxalase Activity during Aging

**Figure 6:**
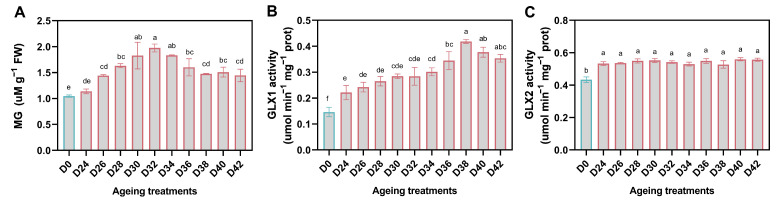
The change in MG content (**A**) and Glyoxalase activity (**B**,**C**) in oat seeds subjected to aging treatment. The letters represent statistical significance among the treatments and the vertical bars represent the ±SEM at *p* < 0.05 level for three replicates. The mean values sharing same letters, obtained from Duncan test, are not different significantly.

MG content in aged seed samples was significantly higher than that in control (D0), except for D24. Within the aging duration, the content of MG increased and reached the peak in D32, and then decreased (Figure 6A). The activities of GLX1 and GLX2 in aged seeds were also significantly higher than those in control seeds. The trend of GLX1 activity increased from 24 to 38 days and maintained higher level, while the activity of GLX2 remained stable during 42-days treatment (Figure 6B,C).

### 3.6. Expression of Genes Acting on ROS Detoxification

**Figure 7:**
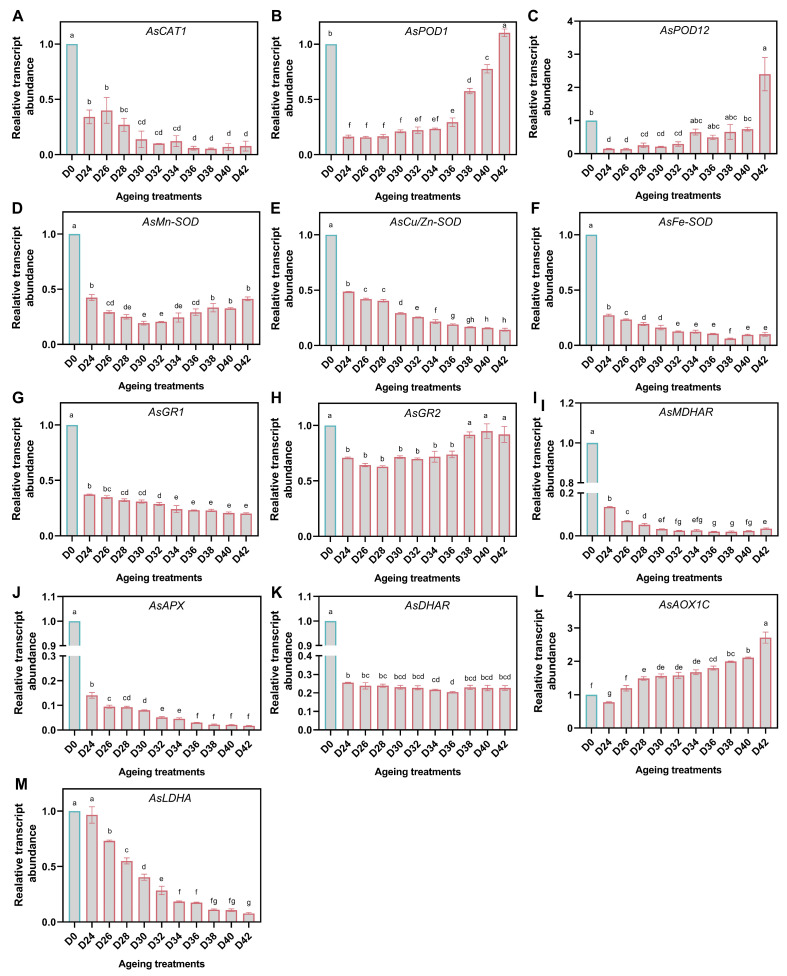
Relative expression levels of genes associated with the ROS scavenging enzymes (**A**–**K**), alternative respiratory (**L**), and anaerobic glycolysis (**M**) in oat seeds. The letters represent statistical significance among the treatments and the vertical bars represent the ±SEM at *p* < 0.05 level for three replicates. The mean values sharing same letters, obtained from Duncan test, are not significantly different.

**Figure 8:**
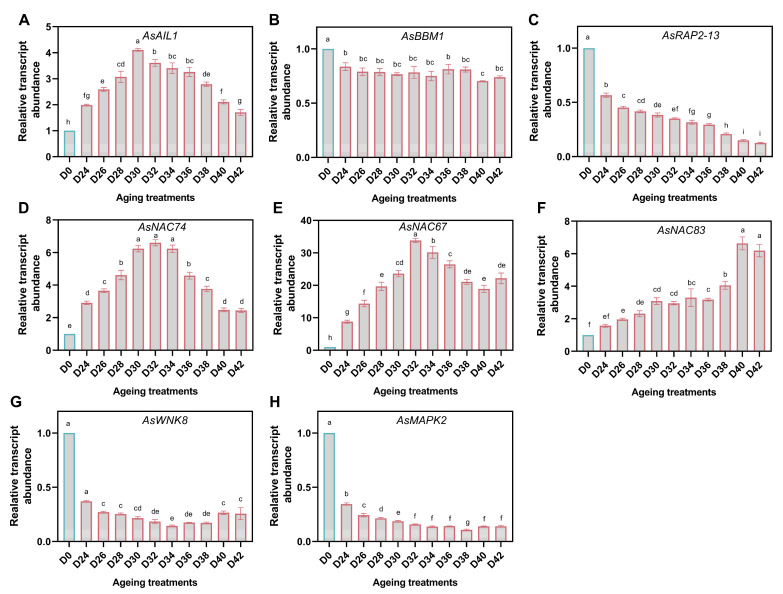
Relative expression levels of AP2 transcription factor family members (**A**–**C**), NAC transcription factor family members (**D**–**F**) and MAPK cascades members (**G**,**H**) in oat seeds. The letters represent statistical significance among the treatments, and the vertical bars represent the ±SEM at *p* < 0.05 level for three replicates. The mean values sharing the same letters, obtained from Duncan test, are not significantly different.

In general, antioxidant defense genes showed diverse patterns during aging, and most of them were down-regulated compared to D0, except for *AsPOD1* and *AsPOD12*, which were up-regulated in D42. More specifically, the abundance of *AsCAT1* showed overall downward trend through aging treatment, and reached its lowest level on the 42nd day after aging (Figure 7A). However, the expression level of *AsPOD1* and *AsPOD12* was higher in D0 and D42, and increased progressively during 42 days of aging (Figure 7B,C). The abundance dynamics of SOD genes varied during aging, as those genes showed different subcellular localization. Within aging duration, the abundance of *AsMn-SOD* gradually decreased to a minimum level at D30, and then increased slowly, while *AsCu/Zn-SOD* and *AsFe-SOD* decreased continuously (Figure 7D–F). Likewise, *AsGR1* and *AsGR2*, localized in different subcellular, also showed different patterns. *AsGR1* decreased gradually, while *AsGR2* initially decreased and then increased with the aging duration (Figure 7G,H). Compared to D0, the abundance of *AsMDHAR* dropped sharply, but there was a little pick-up in aged seed samples (Figure 8I). Similarly, *AsAPX* levels were considerably reduced in aged seed samples (Figure 8J) in comparison to the control of D0, and less *AsAPX* expression was found in severely aged seeds. We observed significant reduction of *AsDHAR* contents in aged seed samples, compared to D0 (Figure 8K), but no further reduction was found in severely aged seeds. In addition, the level of *AsLDHA* was constantly down-regulated, but *AsAOX1C* was up-regulated during aging, except for D24 (Figure 7L,M), which might indicate that alternative respiratory could be more active in response to seed aging treatment than anaerobic glycolysis.

### 3.7. Response of TFs and MAPK Cascades

There are three expression patterns presented in AP2/ERF family members, *AsAIL1*, *AsBBM1*, and *AsRAP2-13* (Figure 8A–C). The abundance of *AsAIL1* was significantly up-regulated in aged seeds, showing a gradual increase, and reached its peak in D30, with a decline trend following. A slightly decrease in *AsBBM1* abundance was observed in aged seed samples, while changes in *AsRAP2-13* were dropped more pronouncedly and sharply within the aging duration. In agreement with the observed patterns of AsAIL1, the expression of *AsNAC74* and *AsNAC67* also increased to a peak in D32, and then decreased. However, compared to *AsNAC74*, *AsNAC67* showed more upregulated expression. Moreover, the abundance of *AsNAC83* increased continuously, and showed the highest level in the most seriously aged seed samples (Figure 8D–F).

MAPK cascades, which were activated by ROS, were reported to account for the antioxidant defense machinery in stress and germination. Interestingly, the expression levels of WNK8 and MAPK2 in aged seed samples decreased significantly compared to D0 (Figure 8G,H). However, the expression level increased in the severely aged seeds which accumulated high level of H_2_O_2_.

### 3.8. Response of GSH-Dependent Glyoxalase Genes

**Figure 9:**
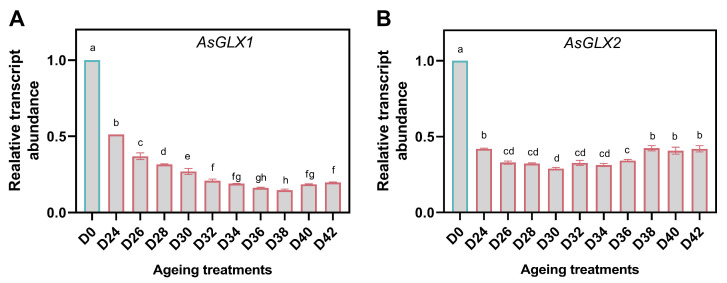
Relative expression levels of GSH-dependent glyoxalase genes, *AsGLX1* (**A**) and *AsGLX2* (**B**) in oat seeds. The letters represent statistical significance among the treatments, and the vertical bars represent the ±SEM at *p* < 0.05 level for three replicates. The mean values sharing the same letters are not different significantly, based on Duncan test.

Two genes, *AsGLX1* and *AsGLX2*, acting on MG detoxification, were down-regulated in aged seeds, and their minimum relative expression values appeared in D38 and D30, respectively. Both of them showed down- and up-regulation patterns within the aging duration, but *AsGLX1* exhibited a more pronounced downward trend than *AsGLX2* (Figure 9A,B).

### 3.9. Correlation Analysis and Change Patterns of 48 Indicators

**Figure 10:**
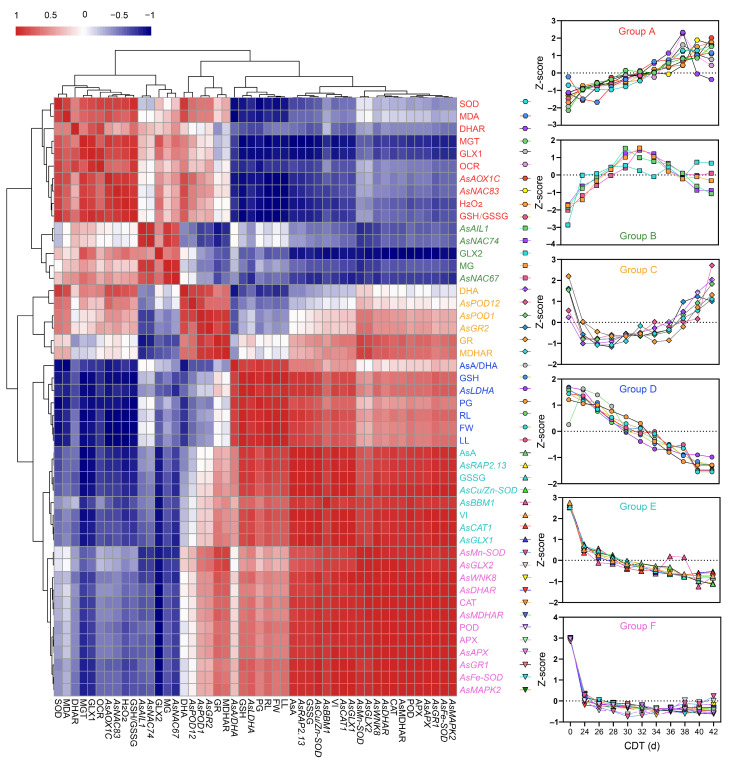
The correlation coefficients between germination indexes, oxygen consumption, ROS and MG content, antioxidants content, antioxidant enzymes activity, glyoxalase activity, and their associated genes transcriptional expression level. According to the correlativity, all 48 indicators were divided into six groups, Group A, Group B, Group C, Group D, Group E, and Group F, which are highlighted with red, green, orange, blue, turquoise, and purple, respectively.

Correlation analysis of the 48 indicators showed there was a high correlation among most of the indicators. Germination percentage (GP) was strongly positively correlated with LL, RL, FW, GSH content, AsA/DHA ratio, and *AsLDHA* abundance, but highly negatively correlated with MGT, ROS content (H_2_O_2_), MDA, OCR, activities of GLX1 and DHAR, GSH/GSSG ratio, and expressions of *AsAOX1C* and *AsNAC83*. Vigor index as a representative indicator of seed vigor was highly correlated with seven indicators, including AsA and GSSG contents, and the expressions of *AsRAP2.13*, *AsCu/Zn-SOD*, *AsBBM1*, *AsGLX1*, and *AsCAT1*. Overall, MDA content and AsA/DHA ratio showed the largest negative correlation (r = −0.976), while APX activity and *AsAPX* expression were with largest positive correlation value of r = 0.999 (Figure 10).

In addition, according to the correlation analysis, 48 indicators were divided into six groups, which showed varied change patterns. Group D with seven indicators, i.e., PG, FW, LL, RL, GSH, AsA/DHA, and *AsLDHA* expression, showed a steady downward trend, and were consistent with the aging process (Figure 10). Contrary to Group D and the descending trend of germination percentage and seedling growth in aged oat seeds, Group A showed a gradual upward trend and contained ten indicators of MGT, SOD, MDA, H_2_O_2_, DHAR, GLX1, OCR, GSH/GSSG, *AsAOX1C* expression, and *AsNAC83* expression. Group B, including MG, GLX2, *AsAIL1* expression, *AsNAC74* expression, and *AsNAC67* expression, had a peak in D30 or D32. On the contrary, Group C showed a considerable decline at the beginning, and then increased with aging time, which contained GR, MDHAR, *AsPOD1*, *AsPOD12*, and *AsGR2*. In Group E (turquoise in Figure 10) and F (purple in Figure 10), a more precipitous decline was observed, compared to Group D.

### 3.10. Cluster Analysis of the Tested Seed Samples

**Figure 11:**
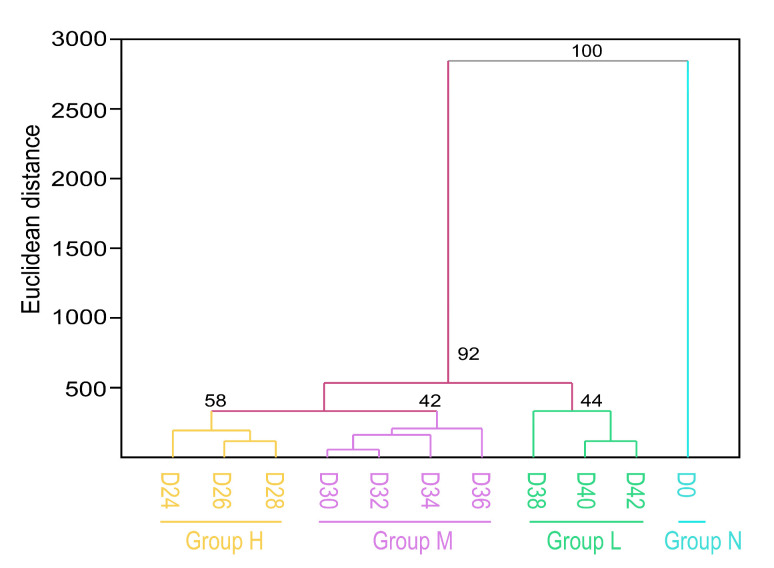
UPGMA clustering diagram based on Euclidean distance of 48 indicators. All eleven oat seed samples were classified into three distinct groups, non-aged group (Group N), higher vigor group (Group H), medium vigor (Group M), and lower vigor group (Group L).

Based on 48 indicators, eleven seed samples were clustered into four groups according to the Euclidean distance. The first group containing only the non-aged sample (D0, GP = 100%) was marked as Group N. The second group including D24, D26, and D28 showed higher vigor (GP from 82.00% to 93.33%) and was marked as Group H. The third group Group M contained seed samples of D30, D32, D34, and D36, and exhibited a medium vigor level (GP from 19.33% to 73.33%). The fourth group Group L, including D38, D40, and D42, showed lower vigor (GP from 0% to 5.33%) (Figure 11).

## 4. Discussion

### 4.1. The Full-Length Transcriptome Is a Key Genetic Resource for Oat

The comprehensive genetic information obtained through whole genome sequencing and transcriptome sequencing provides access to plant improvement. Polyploid plants, such as oat, wheat (*Triticum aestivum*), and peanut (*Arachis hypogaea*), often showed higher complexity and variability of gene information. Direct single-molecule full-length transcriptome sequencing can generate high-quality gene information economically and efficiently, and can enhance our understanding of the transcriptome complexity, such as AS events and lncRNAs [26,27]. Previous work on oat constructed long-read mRNA libraries from developing seeds through Illumina MiSeq^®^ platform. The Trinity longest isoform set showed an average length of 757 bp, and only 27,278 transcripts were longer than 1000 bp [31]. The present work constructed a full-length mRNA library from pooling mRNA of seed embryo, endosperm, leaf, stem, root, and floret using the PacBio RS system. A total of 107,320 consensus isoforms with an average length of 2094 bp were obtained, and 59,050 non-redundant transcripts were annotated. In addition, we identified 3332 lncRNAs, 19,281 SSRs, and 2164 TFs. EST-SSR markers have the advantages of good transferability and are important tools for molecular assisted breeding [49]. LncRNAs do not encode proteins, but they exert a regulatory effect on multiple biological processes [50]. TFs are essential regulatory proteins, and play important roles in the regulation of gene expressions in response to developmental programs and adverse conditions [51,52,53]. Taken together, the full-length transcriptome data positively facilitated the annotation of the oat genes and could promote further studies on the genetic function.

### 4.2. ROS Generation and Detoxification Are Important in Seed Aging

Seed aging was considered to be mainly caused by the oxidative toxicity of ROS, which may lead to a series of metabolic disorders in the seed, such as energy supply, antioxidant system, hormone signal transduction, MAPK cascades, and transcriptional regulation [3,12,15,54]. Therefore, strict regulatory pathways must be induced to control ROS homeostasis so as to ensure the normal seed germination and seedling growth. Both natural aging and artificial aging treatments would induce an increase in ROS content in the seeds, which has been confirmed in many species [4,55,56]. In oat seeds, the content of H_2_O_2_ gradually increased with the aging duration, which was highly positively correlated with MDA content, but negatively correlated with seedling growth. It suggested that membrane lipid peroxidation in aged seeds is considerable, especially in mitochondrial membrane, which is closer to ROS production in seeds [6,8]. The oxygen consumption rate of imbibed oat seeds also increased, and started to decline until germination percentage decreased to 0%. Previous works have reported that mitochondrial structure was damaged after severely aging, and the increase of oxygen consumption might lead to electron leakage from the electron transport chain, resulting in more ROS accumulation [4,5]. In addition, the increasing expression of *AsAOX1C* suggested that oat seeds might prevent excessive ROS production and alleviate oxidative damage by alternative respiration pathway during aging. However, anaerobic glycolysis acts as another protective strategy against ROS, and serves as a means of energy production [11]. Its main participant of lactate dehydrogenase-B was continuously down-regulated in expression, which might also cause the ROS toxicity.

Seeds have an orderly internal enzymatic and non-enzymatic antioxidant system to cope with aging or adverse conditions [3,6]. GSH and AsA are directly involved in ROS detoxification, and their contents are highly and positively correlated with germination percentage and vigor index in oat seeds. Previous studies have indicated both endogenous and exogenous GSH and AsA were participated in the regulation of seed vigor [4,12,57]. Exogenous GSH and AsA could effectively alleviate aging damage in oat seeds, by enhancing activities of antioxidant enzymes in the AsA-GSH cycle, cytochrome c oxidase, and mitochondrial malate dehydrogenase, and maintaining mitochondrial structures in embryonic cells [4]. In addition, as an indispensable small intracellular thiol molecule, GSH could improve Arabidopsis seed germination, because it was participated in the earliest events in energy and redox metabolism during imbibition, and avoided protein oxidation in mitochondrial respiratory chain and TCA cycle [12]. 

Additionally, we found DHA content increased gradually with the aging duration, indicating that the reduced AsA was converted into the oxidized form, which was consistent with the performance of plants under oxidative stress [58]. However, the change of oxidized GSH was similar to that of its reduced form, but oxidized GSH decreased more rapidly, resulting in an increase of GSH/GSSG. This might be due to the rapid decrease of expression of genes which are involved in ASA-GSH cycle. In addition, CAT, POD, GR, MDHAR, and APX activity were also lower in the aged seeds than those in non-aged seeds. This also might be one of the reasons for the increase of H_2_O_2_ accumulation in aged oat seed. SOD activity in aged oat seed samples showed significant increase, but studies on mung bean and tomato, it showed a gradually decrease under continuous aging [15,59]. 

Additionally, the activity of serval antioxidant enzymes was inconsistent with the gene expression, which might be affected by post-translational modification [60], regulation of inhibitors and activators [61], as well as changing environment [62]. The present study found that the activity of GR and MDHAR, and the transcripts abundance of *AsGR2* and *AsMDHAR* increased after an initial decrease, while *AsGR1* expression decreased continuously. The increased accumulation of ROS in mitochondrial might induce the expression of *AsGR2* and enzyme activity in severely aged seeds. Additionally, GR2 has been suggested to be an important regulator of leaf senescence and seed aging in *Arabidopsis* [12,63]. Hence, we inferred that *AsGR2* might be much involved in regulation of oat seed vigor. 

Several works have revealed the significant response of MDHAR in gene expression and enzyme activity after seed aging [4,15], but the function of MDHAR in seed longevity were still unclear. However, DHAR1, one of the three DHAR members acting on AsA regeneration in the AsA-GSH cycle, has been discovered in *Arabidopsis* by genome-wide association, and its mutant presented less seed longevity [13]. In addition, as a mitochondrial enzyme, Mn-SOD was also induced in the severely aged oat seeds, and its strongest antisense *Arabidopsis* line showed lower seed vigor under control growth conditions [64]. Considering the above-mentioned, it appears that antioxidant defense genes function in mitochondrial protection might be one of the significant factors in oat seed longevity.

### 4.3. MAPK Family and NAC and AP2/ERF TFs Are Potentially Involved in Seed Vigor Modulation

H_2_O_2_ has been confirmed to affect seed germination and seed longevity through activating multiple MAPK cascades which are involved in responses to various biotic and abiotic stresses and developmental processes [15,65,66]. WNK is a newly discovered protein kinase of MAPKKK family that has been reported as a regulator of plant stress resistance and tissue development [67,68], but little is known of their roles in seed biology. In rice, a genome-wide expression analysis of WNK kinase gene family showed that *WNK6*, *WNK7*, and *WNK8* might potentially play a role during heat stress [69]. *Arabidopsis* T-DNA knock-out *wnk8* mutant showed enhanced tolerance to severe salinity and osmotic stresses compared to wild type, and higher activities of CAT and POD might be the potential cause [70]. We found that *AsWNK8* was significantly down-regulated in aged seeds, but there was a slight up-regulation in the severely aged seeds. Its expression level during aging was under high correlation coefficients with the expression of *AsMDHAR*, *AsMn-SOD*, and the activity of CAT. Moreover, previous works indicated that WNK8 was involved in ABA signal transduction and ABA synthesis, and its RNAi line exhibits higher ABA content than that of the wild [67,71]. It is commonly known that ABA inhibits seed germination, so the down-regulation of WNK8 in this study may cause an increase in ABA content, and thus inhibit seed germination. MAPKs has been reported to account for the modulation of the antioxidant defense machinery in stress and germination, but little is known about their function in seed aging. MAPK3 and MAPK6 were suggested to be potential contributors of seed aging, as their expressions were up-regulated during the tomato seed aging in protein levels [15]. However, we found that *AsMAPK2* expression showed the opposite trend to tomato MAPK3 and MAPK6 after aging, which might also be a reason for the decrease of seed vigor caused by the disruption of MAPK cascade signaling.

NAC and AP2/ERF families are commonly involved in various biological processes, including seed germination, biotic and abiotic stress response, and plant senescence [51,52,72]. NAC family members were found to respond to abiotic stresses by regulating plant antioxidant system. For instance, under low temperature stress, the overexpression of *SLNAC1* in potato reduced the accumulation of ROS and maintained the activities of SOD and CAT [73]. Similarly, ROS production and MDA content increased in *CaNAC064*-silenced pepper plants, but the activities of SOD, POD, and CAT significantly increased in the *CaNAC064*-overexpressing *Arabidopsis* plants under low temperature stress [74]. Additionally, *NAC* genes also play critical roles in stress responses and plant senescence, such as *NAC002*, *NAC019*, *NAC055*, *NAC072*, and *NAC083* [53,75]. AP2/ERF family members were also widely involved in response to oxidative damage. For instance, RAP2.4 is a redox-sensor and a transducer of redox information, and it has been revealed to be involved in oxidative tolerance [76]. RAP2.11 expression would be stimulated by ROS under the K^+^-deprived conditions [77]. However, NAC and AP2/ERF family members are rarely reported in seed aging and seed vigor. *CarNAC4*-transgenic plants exhibited more enhanced drought and salt tolerance than the Col-0 plant at germination and seedling stages, and had a lower level of MDA than that of wild type [78]. In rice, triple knockout of the genes *BBM1*, *BBM2*, and *BBM3* seriously affected the seed vigor, and only 2 of 191 seeds germinated, which revealed that BBMs were almost indispensable to maintain the seed vigor [79]. Our findings showed that the expression of *AsBBM1* decreased significantly after aging, which might also be one of the reasons for the loss of oat seed germination capacity. Overall, we characterized the responses of partial members of AP2/ERF and NAC families, which are the potential regulators of seed vigor of oat, under aging dynamics.

### 4.4. Seed Aging Is Accompanied by Disordered Glyoxalase System

Glutathione-dependent glyoxalase pathway has been confirmed to be the primary route for MG detoxification. Overexpression of glyoxalase genes, either individually (GLXI/GLXII) or as a complete pathway (GLXI + GLXII), in plant species such as rice, tobacco, tomato, and Carrizo citrange rootstock has resulted in plants with improved tolerance to salinity, drought, heavy metals, and oxidative stresses [17]. The regulatory model of GLX1 and GLX2 were sophisticated, because both of them showed multiple homologs and alternative splicing derived isoforms which had different subcellular localizations [80]. In *Arabidopsis*, loss-of-function lines showed GLXI;3 isoform could eliminate toxic reactive carbonyl species during seed germination and seedling establishment [80]. Additionally, aldo-keto reductases (AKRs), one of the members of glutathione-independent pathways, have been suggested to be one of the reasons to improve the seed longevity during storage. The transgenic rice and tobacco seeds showed lower accumulation of MDA, MG, and Maillard and Amadori products, which caused inconvenience to the seed viability and germination [81]. The expression levels of *AsGLX1* and *AsGLX2* were highly consistent with vigor index with aging duration. In addition, MG content and the activity of GLX1 and GLX2 were significantly higher than those in non-age seed samples, and increased gradually as the germination percentage decreased. These results were different from the antioxidant system, although both of oxidative damage and MG detoxification were caused by imperfect metabolism. GLX2 convert MG and GSH into S-D-lactoylglutathione in chloroplast and mitochondria, but in imbibition seed embryos, mitochondria were the metabolically active organelle. Moreover, the expression of *AsGLX2* was slightly up-regulated in the most seriously aged seeds. Considering all of the cases, we inferred that *AsGLX2* also might be a potential regulator of oat seed longevity.

### 4.5. Promising Indicators in Seed Germination and Seed Age Evaluation

The prediction of seed germination and seed age is valuable for oat germplasm conservation and the seed industry. In agricultural production, it is necessary to ensure that the seeds have high germination percentage, good consistency, and strong resistance [1,2]. In addition, oat seeds contain diverse essential nutrients, which can deteriorate gradually with the duration of storage [23]. We found that the content of GSH, the AsA/DHA ratio, and *AsLDHA* expression were highly correlated with germination percentage and seedling growth, while AsA and GSSG contents, and expressions of *AsCU/Zn-SOD*, *AsCAT1*, *AsGLX1*, *AsBBM1*, and *AsRAP2-13* were consistent with seed vigor and aging days. In the future, these indicators can be potentially applied in predicting and determining seed germination and seed age in the oat seed industry, especially being valuable in sowing and commercial transaction.

## 5. Conclusions

**Figure 12:**
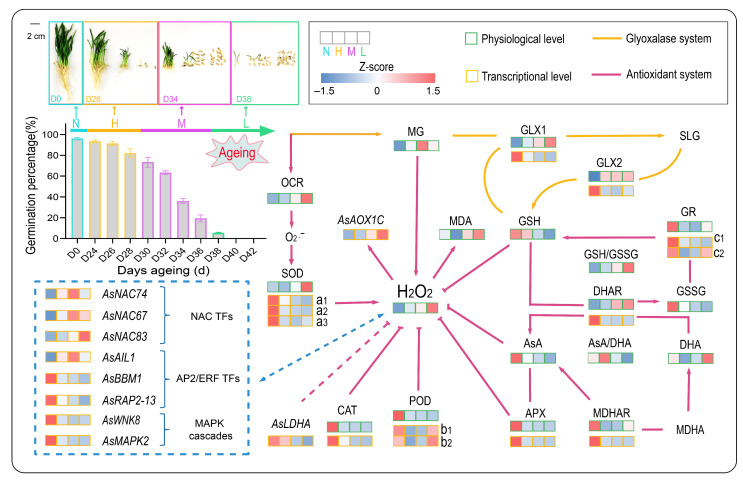
Putative physiological and transcriptional response model for varying degrees of aging in oat seeds. N, H, and L represent three vigor level of groups, non-aged group (Group N), high vigor group (Group H), and low vigor group (Group L), respectively. Orange and green rectangles represent changes in transcriptional expression level and physiological level, respectively. All the data of 48 indicators was normalized (Z-score) based on the mean and standard deviation. The lowercase characters represent genes of *AsCu/Zn-SOD* (a1), *AsFe-SOD* (a2), *AsMn-SOD* (a3), *AsPOD1* (b1), *AsPOD12* (b2), *AsGR1* (c1), and *AsGR2* (c2).

The present study constructed a well annotated comprehensive full-length transcriptome of oat using the PacBio RS system. Based on the annotated transcripts, we revealed the transcriptional and physiological dynamic responses of both the antioxidant system and the glyoxalase system during seed aging. All 48 indicators showed six response patterns, and the levels of GSH, AsA/DHA ratio, and *AsLDHA* abundance might be promising indicators for seed germination and seedling growth. We proposed a putative seed aging response model for the four groups of non-aged group (Group N), higher vigor group (Group H), medium vigor (Group M), and lower vigor group (Group L) (Figure 12). Mild and moderate aging treatments resulted in decreases of contents of almost all antioxidants, expression of genes related to antioxidant enzymes and glyoxalases, and activity of most antioxidant enzymes, and moderate aged seeds showed more decrease than mild ones. Mild aged seeds maintained relatively higher GSH content and ASA/DHA ratio, while moderate aging caused rapid accumulation of H_2_O_2_, MG, and MDA, which considerably reduced seed germination percentage. Meanwhile, *AsAOX1C* expression and the activities of DHAR, GLX1, and GLX2 were significantly up-regulated to cope with the toxicity caused by excessive accumulation of ROS and MG. In severely aged seeds, most detoxifying enzymes and genes were at their lowest levels, and ROS accumulation and lipid peroxidation were further increased, resulting in failure of germination. Nonetheless, significant increases of expression of *AsPOD1*, *AsPOD12*, *AsGR2*, and activities of SOD, GR, and MDHAR suggested they were still active in ROS detoxification. GLX1 and GLX2 activities also maintained high levels in severely aged seeds, acting on the decrease of MG accumulation. In addition, we found MAPK cascade and TF members significantly responded during oat seed aging, and further studies are needed to better understand their roles in oxidation mediated seed aging. Overall, the present study provided an insight into the pathways and mechanisms underlying the time-course dependent responses of aged oat seeds.

## Figures and Tables

**Table 1:** Summary of PacBio sequencing data in oat.

Data Size (G)	CCS	FLNC Reads	Number of Consensus Isoforms	Mean Read Length of CCS	Number of Polished High-Quality Isoforms	Non-Redundant Full-Length Transcripts
22.24	293,642	244,868	107,320	2094	103,732	67,184

## Data Availability

Data is contained within the article and supplementary material. Raw sequencing data of the full-length transcriptome used in the current study are available in the NCBI’s Sequence Read Archive (SRA, https://www.ncbi.nlm.nih.gov/sra, accessed on 18 November 2021) under the BioProject PRJNA781458.

## Supplementary Materials

The following supporting information can be downloaded at: https://www.mdpi.com/article/10.3390/antiox11020395/s1, Figure S1. Statistics of identified lncRNAs, SSRs and TFs. (A) Venn diagram of identified lncRNAs from the Coding Potential Calculator (CPC), Coding-Non-Coding Index (CNCI), CPAT (Coding Potential Assessment Tool) and Pfam database. (B) Density distribution of seven SSR types. c, compound SSR; p1, mono-nucleotide; p2, di-nucleotide; p3, tri-nucleotide; p4, tetra-nucleotide; p5, penta-nucleotide; p6, hexa-nucleotide. (C) Statistics of the top 20 TFs; Table S1: Details of genes and their primers for qRT-PCR analysis.
