# Supplementary material for: Dynamic Responses of Antioxidant and Glyoxalase Systems to Seed Aging Based on Full-Length Transcriptome in Oat (Avena sativa L.)
Source: Antioxidants (Basel). 2022 Feb 16;11(2):395. doi: 10.3390/antiox11020395 (PMC8869221; doi:10.3390/antiox11020395)
Supplement: Supplementary file 1 [file antioxidants-11-00395-s001.zip › antioxidants-1594399-SI.pdf]

Table S1. Details of genes and their primers for qRT-PCR analysis

No.	Gene code	Accession number	Nr/Nt description	Primer Sequence (5′-3′)	Product Size (bp)	Tm (℃)
1	*AsCAT1*	F01_transcript_13967	*Aegilops tauschii* subsp. strangulata catalase isozyme 1	F: ACAGGGATGAGGAGGTGAACTAC<br>R: CGCTGGATGAAACGGTCTTGC	193	58.8<br>59.7
2	*AsPOD1*	F01_transcript_67131	*Triticum dicoccoides* 1-Cys peroxiredoxin	F: GCCCGACAAGAAGGTGAAGC<br>R: GCCGTGAGCAGCGAGTCC	96	58.6<br>59.9
3	*AsPOD12*	F01_transcript_17118	*A. tauschii peroxidase 12*	F: CCCAAGGCAGAGTCCATCGTG<br>R: CCGTCCAGGAGCACTGAAGC	137	60.3<br>60.1
4	*AsMn-SOD*	F01_transcript_21558	*Stipa purpurea* Mn-superoxide dismutase	F: AGAAGCACCACGCCACCTAC<br>R: CGCCGCCGTTGAACTTGATG	126	59.4<br>59.9
5	*AsCu/Zn-SOD*	F01_transcript_22817	*Agrostis stolonifera* cytosol Cu/Zn-superoxide dismutase	F: ACCGCCGTGACAGGAAGC<br>R: GCAACACCATCCGCTCCAG	200	59.1<br>58.1
6	*AsFe-SOD*	F01_transcript_33161	*A. tauschii* subsp. strangulata Fe-superoxide dismutase	F: GCTGATGTGCTTGAATCCTTGAAC<br>R: GTGCTTGTGCTGGTGAACTCC	150	58.8<br>59.3
7	*AsGR1*	F01_transcript_53759	*A. tauschii* subsp. strangulata glutathione reductase, cytosolic	F: AGAACAGCATATCCAAACGACAG<br>R: CAGCCTTGAGTGCGACAGC	142	57.5<br>58.4
8	*AsGR2*	F01_transcript_81228	*T. dicoccoides* glutathione reductase, chloroplastic-like	F: ACGACTACGACCTCTTCACCATC<br>R: GAACACACCCACGAAGCACAC	171	59.4<br>59.5
9	*AsMDHAR*	F01_transcript_29627	*B. distachyon* monodehydroascorbate reductase	F: GCGGAGCAGGCAGTGAAGG<br>R: GCGAGTAGAAGTAGGGCAGGTAG	85	58.4<br>56.5
10	*AsAOX1C*	F01_transcript_105355	*T. dicoccoides* ubiquinol oxidase 1c, mitochondrial-like	F: GCTGGAGGAGGCGGAGAAC<br>R: TCGGTGTAGGAGTGGATGGC	200	59.1<br>58.3
11	*AsLDHA*	F01_transcript_70112	*T. dicoccoides* L-lactate dehydrogenase A-like	F: CCTTGCCACCTCCCTCCTC<br>R: CAGTTCTCCCACAGCGTCTTG	213	58.3<br>58.5
12	*AsGLX1*	F01_transcript_18206	*T. dicoccoides* lactoylglutathione lyase	F: AAGTTATGCTCCGTGTTGGTGAC<br>R: TGCGTTGCCCTTGCTATATTCTG	200	59<br>59.2
13	*AsGLX2*	F01_transcript_67599	*A. tauschii* subsp. strangulata probable hydroxyacylglutathione hydrolase 2	F: CTTGAGCGTAGAACCAGGAAAC<br>R: ATTGTTGTTGGCACCGTTGG	96	56.8<br>56.9
14	*AsNAC74*	F01_transcript_87068	*B. distachyon* NAC domain-containing protein 74	F: TGCCAAGCCTGCCCTGATATG<br>R: ATTATTGTTGCCCACCTCGTTTCC	102	60<br>59.7
15	*AsNAC67*	F01_transcript_27463	*B. distachyon* NAC domain-containing protein 67	F: CCGCCTTGCCGACACCTC<br>R: TCTTCTCCCATTCGTTCTTCTTGC	107	59.8<br>59
16	*AsNAC83*	F01_transcript_44175	*B. distachyon* NAC domain-containing protein 83	F: CGGAAGGCTGGGTGCTCTG<br>R: TCGTGGACGGGATCTTCTTGG	118	59.7<br>59
17	*AsAIL1*	F01_transcript_28993	*A. tauschii* subsp. strangulata AP2-like ethylene-responsive transcription factor AIL1	F: GACGAGACAGGAGTTCATTGCG<br>R: TGCCGTGCTGGTGATGCC	101	59.1<br>59.8
18	*AsBBMI*	F01_transcript_63832	*A. tauschii* subsp. strangulata AP2-like ethylene-responsive transcription factor BBM1	F: CCACCAGTATCTGCCTCCACAAC<br>R: TTCGTCCACTTCCTCATTCTTCGG	147	60.7<br>60.5
19	*AsRAD2-13*	F01_transcript_77110	*A. tauschii* subsp. strangulata ethylene-responsive transcription factor RAP2-13	F: CCGCCGCAGCAGAGGTTC<br>R: GCGAGAAGCGAGTCCCAGTC	124	60.2<br>59.9
20	*AsWNK8*	F01_transcript_61751	*A. tauschii* subsp. strangulata probable serine/threonine-protein kinase WNK8	F: CGTGGAGGTGGCGTGGAAC<br>R: GGCGTGGAAGGCGATGATGG	130	60.2<br>60.9
21	*AsMAPK2*	F01_transcript_37493	*T. aestivum* mitogen-activated protein kinase 2	F: ATAAGTGATACTCCAACTGCCATC<br>R: TATCTATTGCTGGTGTGTACTTCG	112	56.2<br>56.3
22	*AsAPX*	F01_transcript_31727	*Puccinellia tenuiflora* ascorbate peroxidase	F: AAGGACTATGCTGAATCACACAAG<br>R: CAACTGCTACGCCGACTGC	133	56.9<br>58.5
23	*AsDHAR*	F01_transcript_51051	*P. tenuiflora* dehydroascorbate reductase	F: AAGACCAAGCCAGCCAAGGAG<br>R: AAGGACAGGACAGAACACAGACG	111	59.8<br>60.0
24	*AsEIF4A*	F01_transcript_15006	*B. distachyon* eukaryotic initiation factor 4A-III homolog A	F: TCTCGCAGGATACGGATGTCG<br>R: TCCATCGCATTGGTCGCTCT	88	63.3<br>63.6

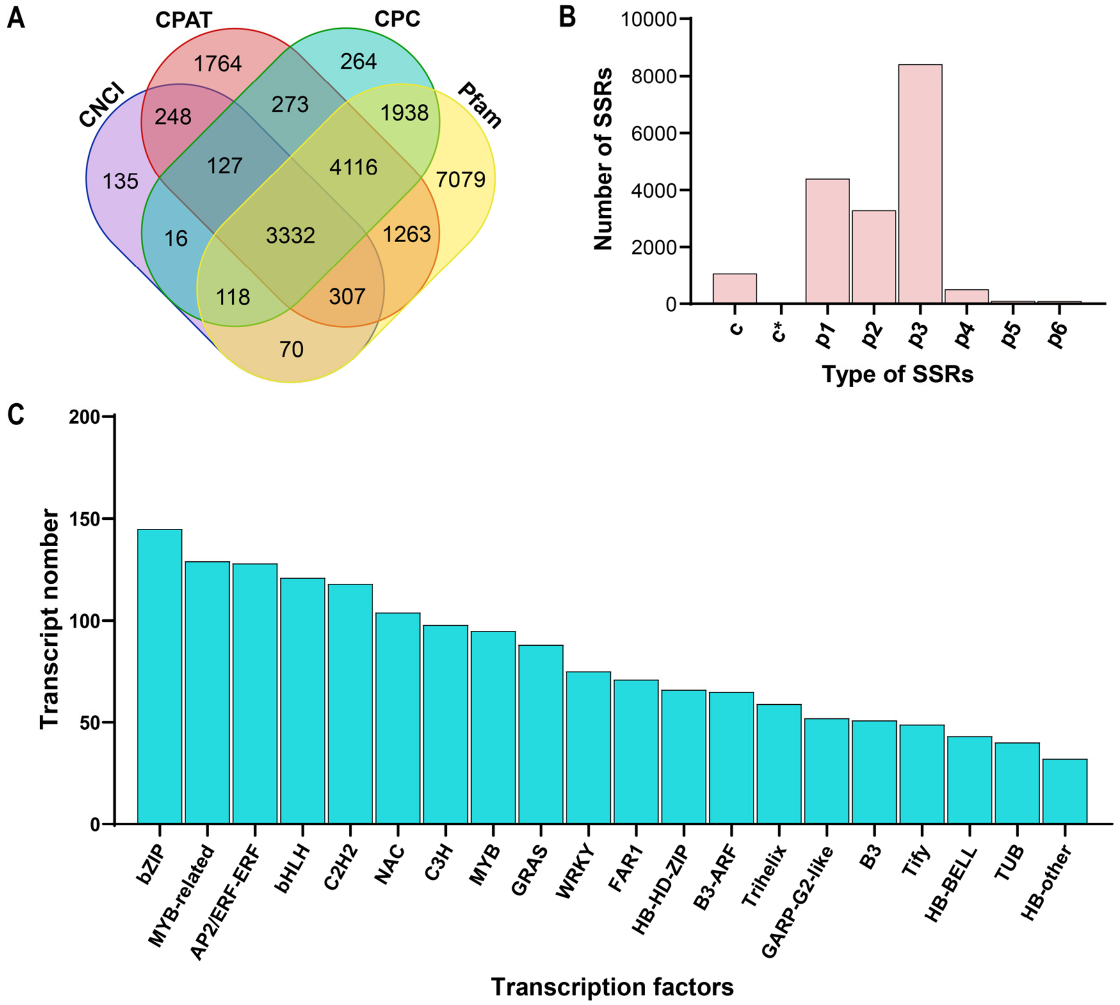

Figure S1. Statistics of identified lncRNAs, SSRs and TFs. (**A**) Venn diagram of identified lncRNAs from the Coding Potential Calculator (CPC), Coding-Non-Coding Index (CNCI), CPAT (Coding Potential Assessment Tool) and Pfam database. (**B**) Density distribution of seven SSR types. c, compound SSR; p1, mono-nucleotide; p2, di-nucleotide; p3, tri-nucleotide; p4, tetra-nucleotide; p5, penta-nucleotide; p6, hexa-nucleotide. (**C**) Statistics of the top 20 TFs; Table S1: Details of genes and their primers for qRT-PCR analysis.
